# Can Continuous Positive Airway Pressure Reduce the Risk of Stroke in Obstructive Sleep Apnea Patients? A Systematic Review and Meta-Analysis

**DOI:** 10.1371/journal.pone.0146317

**Published:** 2016-01-05

**Authors:** Yeshin Kim, Yong Seo Koo, Hee Young Lee, Seo-Young Lee

**Affiliations:** 1 Department of Neurology, Kangwon National University College of Medicine, Chuncheon-si, Gangwon-do, Korea; 2 Department of Neurology, Korea University College of Medicine, Seoul, Korea; 3 Center for Preventive Medicine and Public health, Seoul National University Bundang Hospital, Seongnam, Gyeonggi-do, Korea; Osaka University Graduate School of Medicine, JAPAN

## Abstract

**Background and Purpose:**

Obstructive sleep apnea (OSA) has been shown to increase the risk of stroke. Although continuous positive airway pressure (CPAP) is considered the treatment of choice for OSA, whether treating OSA with CPAP reduces the risk of stroke remains unclear. We aimed to evaluate the effects of CPAP on incidence of stroke in patients with OSA.

**Materials and Methods:**

We conducted a systematic review and meta-analysis of all published studies that provided the number of incident strokes in OSA patients in light of their treatment status with CPAP.

**Results:**

We identified 8 relevant studies: one randomized controlled study (RCT), 5 cohort studies, and 2 studies using administrative health data. The two overlapping cohort studies in women and the elderly and the 2 studies using administrative health data had analyzed the impact of CPAP on stroke apart from cardiac events, whereas the others had focused on the overall cardiovascular events. Based on a meta-analysis of the cohort studies, treatment with CPAP was associated with a lower incidence of stroke and cardiac events with relative risks of 0.27 [0.14–0.53], and 0.54 [0.38–0.75], respectively, although this could not be reproduced in the RCT and the studies using administrative data.

**Conclusions:**

Treating with CPAP in patients with OSA might decrease the risk of stroke, although there is some conflicting evidence. Such effect was more pronounced in stroke than in cardiac events. Future studies analyzing stroke apart from cardiac disease would be of interest.

## Introduction

It is well-known that obstructive sleep apnea(OSA) is associated with cardiovascular diseases (CVD) such as hypertension, arrhythmia, heart failure, coronary artery disease and stroke [1]. Continuous positive airway pressure (CPAP) is currently the most effective treatment for OSA [2], and has been shown to decrease blood pressure among those with OSA [3]. However, whether or not CPAP can improve cardiovascular outcomes remains unclear [4]. A bidirectional relationship between OSA and CVD has been suggested, with OSA merely being an epiphenomenon of CVD [5]. Moreover, one cannot assume treating OSA with CPAP alone would decrease the impact of OSA on CVD, especially in cases of long-standing OSA.

In terms of stroke, OSA increases the risk of stroke and the association is stronger than in other CVD [6–9]. This might be due to underlying mechanisms which are unique to both stroke and CVD. Therefore, treating OSA might impact stroke differently compared to other CVD.

The effects of CPAP after an acute stroke had been recently reviewed [10, 11]. The 2014 AHA/ASA guidelines [10] suggested that treatment with CPAP might be considered for patients with acute ischemic stroke or TIA and sleep apnea, based on improved short-term functional outcomes, although a systematic review pointed out a lack of evidence with regard to the recurrence of cardiovascular events (CVE) [12]. Out of 3 studies which addressed the effects of CPAP on CVE [11], one RCT showed a delay in the appearance of CVE, although the incidence of CVE was not different over 24 month follow-up period [13], and a cohort study reported a significantly lower incidence of CVE in the CPAP group after a 7 year follow-up period [14], whereas the other cohort study lacked power to demonstrate any effects due to low levels of adherence [15].

As for primary prevention of stroke, the effects of CPAP have not been systematically reviewed except the AHA/ASA guidelines on the primary prevention of stroke (2011) which reported that there were no prospective studies on this topic [16].

Therefore we investigated whether CPAP for OSA reduces the risk of stroke based on a systematic literature review and meta-analysis. Our secondary objectives were to compare the effects with those in cardiac events and to identify factors that may affect the effects of CPAP, such as severity, age, sex, and level of adherence.

## Methods

The review of literature was performed according to the Preferred Reporting Items for Systematic Review and Meta-analysis (PRISMA) statement [17].

### Information sources and search methods

We reviewed data using MEDLINE (January 1, 1976 to July 31, 2015), EMBASE (January 1, 1985 to July 31, 2015), and the Cochrane Library (January 1, 1987 to July 31, 2015). All peer review articles regardless of the study design were included.

The search terms that we used were Stroke, OSA and CPAP. We searched for keywords and MeSH related to each theme and combined these using Boolean operator “and” through MEDLINE. Details of the search method are outlined in S1 Table. Search methods for other databases were adapted based on the search methods for MEDLINE. After the initial electronic search, we retrieved relevant articles and bibliographies from the studies identified. Such articles that were identified were individually assessed for inclusion in the study.

### Study selection

The inclusion of a particular study was independently determined between two reviewers based on the selection criteria. Studies were selected via 2 levels of screening: At the first level, we screened the titles and the abstracts of the identified studies. Eligibility was considered if they addressed cardiovascular outcomes of OSA. At the second level, we screened the full manuscript. Studies were included in the systematic review if stroke or cardiovascular outcomes were compared between treated and untreated groups. Studies which focused on outcomes after acute stroke or CVE, or did not comment on stroke as an outcome specifically were excluded.

### Data items

We extracted data from the studies selected using a data extraction form as follows: the first author, the year of publication, the country where the study conducted, study design, the number of participants in treated and untreated groups, sex (% men) of the participants, age, inclusion criteria for OSA, intervention, adherence, follow-up duration, incidence and mortality from stroke, cardiac events, and source of funding.

### Assessment of methodological quality

Two reviewers independently assessed the methodological qualities for each study using the Cochrane risk of bias for RCT [18] and RoBANS [19] for observational studies. Any unresolved disagreements between the two reviewers were resolved through discussion or with a review from a third reviewer. The risk of bias for each study was assessed at the study level and the information was not used in data synthesis.

### Statistical analysis

We specified the number of each stroke, cardiac events and overall CVE (the sum of both stroke and cardiac events), and calculated the relative risk (RR) as the primary outcome. We used Review Manager (RevMan) software version 5.2 for the analysis. A meta-analysis was performed on the cohort studies which provided the number of outcomes of interest, while excluding cohort studies with overlapping participants, RCTs, and studies using health administrative data. To estimate heterogeneity, we estimated the proportion of between-study inconsistency due to true differences between studies, rather than differences due to random error or chance, using the *I*^2^ statistic, with values of 0 to 40%, 30 to 60%, 50 to 90%, and 75 to 100% considered to represent insignificant, moderate, substantial and marked levels of heterogeneity, respectively [20]. Q statistics were also obtained and we considered p<0.05 as significant heterogeneity. A Forest plot was generated for each meta-analysis.

## Results

### Identification and characteristics of the studies

**Fig 1:**
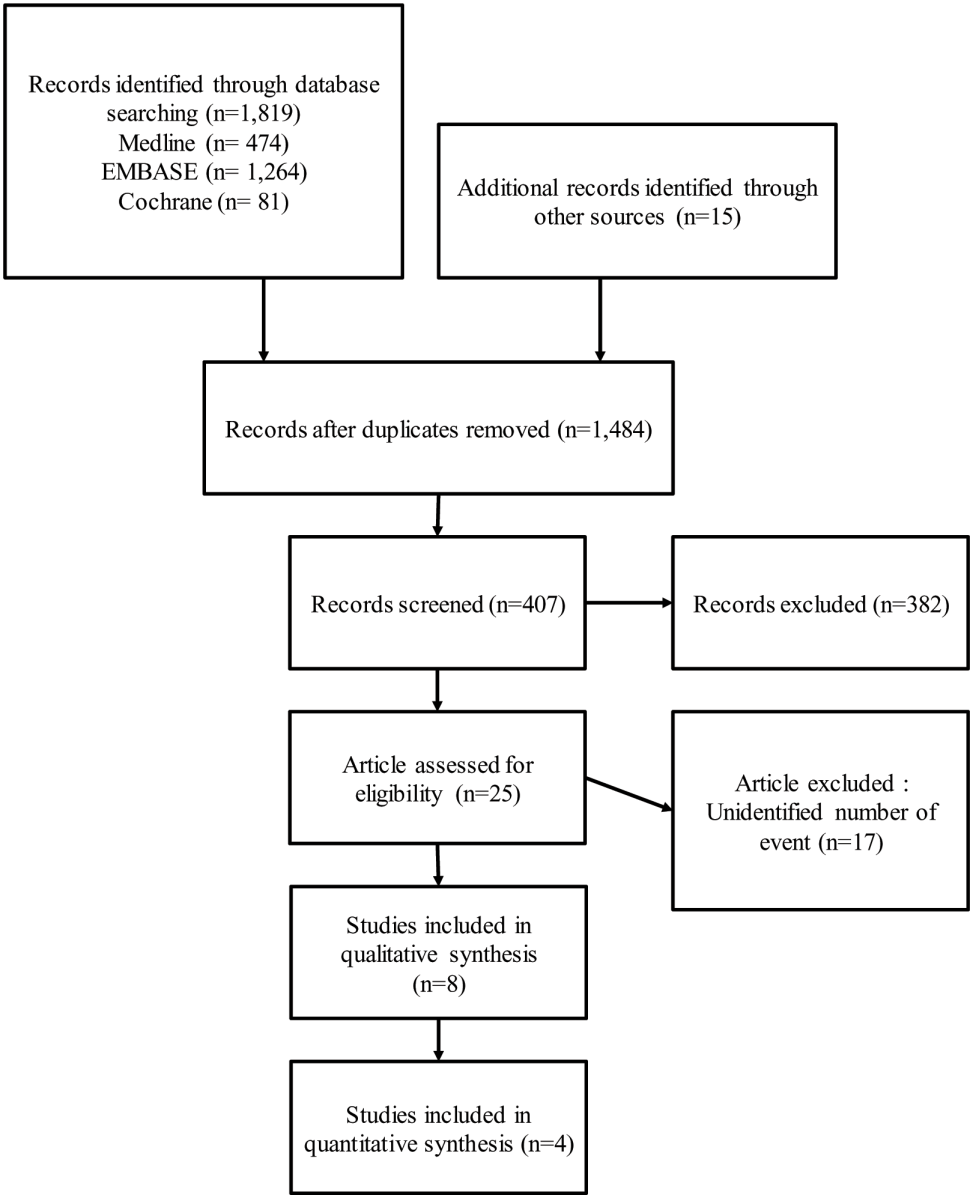
PRISMA flow diagram. Flow diagram demonstrating the process of article selection for systematic review and meta-analysis.

Ultimately we identified 8 relevant articles from 6 study groups: a RCT [21], 5 cohort studies [22–26], and 2 studies using administrative health data (Fig 1) [27, 28]. Three studies from the same cohort reported different outcomes such as incidence [26] and mortality [24, 25] in different subgroups such as women [24, 26] and the elderly [25] respectively, with overlap of participants. Four of the cohort studies were conducted prospectively [23–26], and the fifth was conducted mostly prospectively including small number of retrospectively recruited subjects [22]. The other two studies retrospectively analyzed health administrative data- Danish National Patient Registry records [27] and VA Inpatient and Outpatient Medical SAS data for US veterans [28].

The number of subjects ranged from 168 to 33,274, which was a total of 60,186 subjects, which consisted of 18,293 treated and 41,893 untreated patients with OSA.

Two studies from a same cohort included only women [24, 26] while the others consisted of predominantly men (60~96%). The age of the subjects mostly ranged from their 40s to 60s, except for one cohort study, which included only elderly patients (aged 65 and above) [25]. Patients with previous CVD were excluded in the RCT study [21] and one of the cohort studies [26] but was included in the other 6 studies [22–25, 27, 28].

Diagnosis of OSA and recommendation of CPAP was based on sleep study in the RCT [21] and 5 cohort studies [22–26], whereas the 2 studies which used administrative data identified OSA patients based on their ICD-codes [27, 28]. The apnea-hypopnea index (AHI) criteria for diagnosis of OSA and CPAP treatment varied from 5 to 20 events/ hour.

In the study by Buchner et al, subjects were treated with CPAP (81.3%), BiPAP (13.1%) and intraoral appliance (5.5%) with analysis of the outcome being performed for entire modality [23], whereas CPAP was used exclusively in the rest of the studies. The three cohort studies which involved women [24, 26] and the elderly [25] regarded those who used CPAP for less than average 4hour as being untreated, whereas the others considered all CPAP patients as being treated regardless of adherence.

The duration of follow-up was 48 months for the RCT [21], 72–89 months for the cohort studies [22–26], and 72–132 months for the studies which used the administrative database [27, 28].

Seven studies were conducted in Europe and one in the US [28]. Details of all the studies included are summarized in Table 1.

**Table 1:** Study design, patient characteristics and outcome of interest.

Study (author, year, county	Study designs	Participants (n)	Sex (% men)	Mean age (SD)	Inclu-sion	Treated (n)	Adherence^§^ (%)	Untreated (n)	Reason of no treatment	F/U (m)	Outcomes of interest
Barbe, 2012, Spain [21]	RCT	723	85.6	Treated: 51.8±11.01, untreated: 52.0±10.90	AHI≥20 without EDS	357	64.4	366	Randomized	48	Incidence of new hypertension and overall CVE
Campos-Rodriguez 2014, Spain [26]	Prospective cohort	967	All women	Treated: 58(52–66), untreated: 59(49.2–67.7)*	AHI≥10	441	100^†^	268	Not prescribed** or the patient decline/could not tolerate or adherence<4h/d	80	Incidence of stroke and CHD
Campos-Rodriguez 2012, Spain [24]	Prospective cohort	1116	All women	AHI 10–29 & treated: 58.3±9.8, AHI >30 & treated: 59.1±11.1, AHI 10–29 & untreated: 58.2±12.0, AHI >30 & untreated: 64.2±11.4	AHI≥10	576	100^‡^	262	Not prescribed** or the patient decline/could not tolerate or adherence<4h/d	72	Mortality from overall CVE
Martinez-Garcia,2012, Spain [25]	Prospective cohort	939	AHI<15 60, AHI 15–29& Untreated 65.7, AHI>30&Untreated 71.7, Treated;62.2	Treated: 70.1± 4.2, AHI 15–29 & untreated: 71.7± 5.2, AHI>30 & untreated: 71.9± 4.5	AHI>15	503	100^†^	281	Not prescribed** or the patient decline/could not tolerate or adherence<4h/d	69	Mortality from stroke, heart failure and myocardial infarction
Buchner, 2007, Germany [23]	Prospective cohort	449	85.5	Treated: 55.4±10.5, untreated: 57.8±10.2	AHI≥5	364	78.5	85	Refuse treatment	72	Incidence of overall CVE
Doherty, 2005, Irland [22]	Prospective cohort, mostly	168	92.3	Treated: 50.1±11.4, untreated: 52.8±9.6	AHI>15	107	Not mentioned	61	Never tolerated or stopped for >5yrs	89	Incidence of new hypertension and overall CVE
Molnar, 2015, USA [28]	Retrospective cohort	23,242	Treated 96, Untreated 96	Treated: 57±10, untreated: 59± 11	ICD9-CM: 3272*, CPT codes: 78057	1,478	Unknown	21,764	Unknown	93	Incidence of stroke and CHD
Lamberts, 2014, Denmark [27]	Retrospective cohort	33,274	79	Total OSA men: 52.5±11.8, total OSA women: 54.2±12.0 treated men: 54.4±11.2, treated women:56.5±11.0	ICD-10 code: G473	1,4468	Unknown	18,806	Unknown	132	Incidence of ischemic stroke and myocardial infarction

OSA, obstructive sleep apnea; RCT, randomized controlled study; SD, standard deviation, CPAP, continuous positive airway pressure; AHI, apnea-hypopnea index; CVE, cardiovascular events; EDS, excessive daytime sleepiness; CHD, coronary heart disease

* Mean age(Interquartile range, IQR)

** Criteria for prescription: AHI≥ 30, regardless of symptoms, and AHI 10–29 with EDS>10

^§^Adherence was defined as usage of CPAP for at least 4 hours per night on average.

^†^Adherence among overall CPAP treated patients was not described; only those who used CPAP for 4hours per night or longer were regarded as being treated.

^‡^Adherence among overall CPAP treated patients was 73.6%; only those who used CPAP for 4hours per night or longer were regarded as being treated.

### Quality of the studies (Risk of bias)

The quality of the studies is summarized in Table 2. The RCT had a low risk of bias [21]. In cohort studies, the untreated group consisted of individuals who refused or could not tolerate CPAP, or were not prescribed CPAP at all because of the lack of EDS in mild to moderate cases, which indicates potential selection bias [22–26]. The degree of OSA at baseline was significantly more severe in the treated group in four studies [21, 23, 24, 26] as well as the degree of EDS in the female cohort [24, 26]. Otherwise, the baseline differences between the treated and untreated groups were seldom and adjusted.

**Table 2:** Assessment of bias.

Study design	Study (author, year)	Sequence generation	Allocation concealment	Blinding of participants and personnel	Blinding of assessors	Incomplete outcome data	Selective outcome reporting	Other sources of bias
RCT*	Barbe, 2012 [21]	Low	Low	Low	Low	Low	Low	Low
Study design	Study (author, year)	Selection of participants	Confounding variables	Measurement of exposures	Blinding of outcome assessment	Incomplete outcome data	Selective outcome reporting	
non RCT^†^	Campos-Rodriguez, 2014 [26]	Low	Low	Low	Low	Low	Low	
	Campos-Rodriguez, 2012 [24]	Low	Low	Low	High	Low	Low	
	Martinez-Garcia, 2012 [25]	Low	Low	Low	High	Low	Low	
	Buchner, 2007 [23]	Low	Low	Low	High	Low	Low	
	Doherty, 2005 [22]	High	Low	Unclear	Unclear	High	Low	
	Monlar, 2015 [28]	High	High	High	Unclear	High	Low	
	Lamberts, 2014 [27]	High	High	High	Unclear	High	Low	

*Cochrane Risk of Bias (Randomized controlled study)

^†^RoBANS(Risk of Bias for Nonrandomized Studies)

Studies using the Danish National Patient Registry reported positive predictive values (PPV) of ICD-codes indicating OSA at 82% but did not mention negative predictive values (NPV) [27]. The VA study did not validate the diagnostic performance of these codes for OSA [28]. Moreover, the severity of OSA was not included in both studies [27, 28]. In the validation sample of Danish study, CPAP treated group had more severe OSA than untreated group [27].

In VA study, they considered the possibility of misclassification of treated and untreated group in case of patients who received CPAP treatment outside the VA system [28].

Adherence to CPAP treatment in the included studies which reported adherence [21, 23–26] was within the range of or higher than the adherence generally reported [2]. CPAP adherence data were not available in a cohort study [22] and two studies using administrative DB [27, 28].

Only RCT [21] and one cohort study [26] reported that they were blinded for outcome assessment. Outcomes were assessed by routine outpatient visits in the RCT and by multiple sources of information from clinic visit, reviewing medical records, computerized database, death certificates, the contacting primary physician or telephone calls in the cohort studies. The two studies which used administrative data [27, 28] identified outcomes through diagnostic codes for CVE. This process was validated in the Danish study, but they assessed only the PPV (97%) and their searching criteria could have led to missed events that did not require hospitalization [27]. The VA study did not validate the diagnostic codes for CVE [28].

The RCT [21] and the Danish study [27] were funded by the companies manufacturing the CPAP machine. Publication bias cannot be fully excluded.

### The effects of CPAP on stroke

#### Effects on the incidence of stroke

We identified 6 studies [21–23, 26–28] that provided the number of incident stroke events depending on treatment and the results of individual studies are summarized in Table 3. One cohort study consisting of a women [26] and two studies using administrative data [27, 28] analyzed the effects of CPAP on stroke separately from overall CVE, whereas other studies [21–23] assessed the effects of CPAP on CVE in general. The women cohort study reported untreated OSA was more strongly associated with stroke (adjusted HR 6.44 [1.46, 28.34]) than with coronary disease (adjusted HR 1.77 [0.76, 4.09]), and the risk was normalized in those treated with CPAP for either stroke or coronary disease (adjusted HR 1.31 [0.26, 6.59], 0.70 [0.29, 1.70], respectively), compared with the controls without OSA [26]. However, CPAP was not shown to have effect on the incidence of either stroke or coronary heart disease in two studies using administrative data [27, 28].

**Table 3:** Effects of CPAP on stroke and cardiovascular events.

Study (author, year, country)	Relative risk	Remarks
Barbe, 2012, Spain [21]	Adjusted IDR for hypertension or cardiovascular events, compared with untreated: 0.81(0.61–1.06) in overall treated; 0.69 (0.50–0.94) in adherent group (usage of CPAP for 4hours per night or longer)	
Campos-Rodriguez, 2014, Spain [26]	Adjusted HR for CVE compared with control with AHI<10: 2.76 (1.35–5.62) in untreated versus 0.91 (0.43–1.95) in treated; for stroke: 6.44(1.46–28.3) in untreated versus 1.31 (0.26–6.59) in treated; for CHD: 1.77(0.76–4.09) in untreated versus 0.70 (0.29–1.70) in treated	More risk reduction in stroke than in CHD
Campos-Rodriguez, 2012, Spain [24]	Adjusted HR for cardiovascular mortality compared with control with AHI<10: 3.50(1.23–9.98) in untreated severe versus 0.55 (0.17–1.74) in treated severe, 1.60 (0.52–4.90) in untreated mild to moderate versus 0.19 (0.02–1.67) in treated mild to moderate group	Benefit for overall CVE in only severe group (AHI≥30)
Martinez-Garcia, 2012, Spain [25]	Adjusted HR for cardiovascular mortality compared with control with AHI<15: 2.25 (1.41–3.61) in untreated severe group and 1.38 (0.73 to 2.64) in untreated mild to moderate group versus 0.93 (0.46 to 1.89) in overall treated group; 3.87 (1.12–13.3) in untreated severe group versus 1.01 (0.27–3.36) in overall treated in subgroups of patients 75 years age or older	Benefits in elderly people
Buchner, 2007, Germany [23]	Adjusted HR for CVE compared with untreated: 0.36(0.21–0.62) in overall treated; 0.37 (0.17–0.78) in mild to moderate subgroup	Benefit for overall CVE also in mild to moderate group (AHI 5 to <30)
Doherty, 2005, Ireland [22]	Cardiovascular mortality: 14.8% in untreated versus 1.9% in treated (p = 0.009); Overall CVE: 31% in untreated versus 18% in treated (p<0.05)	
Monlar, 2015, USA [28]	Adjusted HR compared with OSA negative patients, for ischemic stroke: 3.48 (3.28–3.64) in untreated versus 3.50(2.92–4.19) in treated; for CHD: 3.54 (3.10–3.69) in untreated versus 3.06 (2.62–3.56) in treated	
Lamberts, 2014, Denmark [27]	Adjusted IRR, for ischemic stroke: 0.99(0.82–1.19); for myocardial infarction: 0.99(0.85–1.15)	

IDR, incidence density ratio; HR, hazard ratio; IRR, incidence rate ratio; AHI, apnea-hypopnea index; CHD, coronary heart disease; CVE, cardiovascular event; Parentheses indicate 95% confidence interval

On the incidence of overall CVE, RCT could not find significant effect of CPAP. However, a post hoc analysis of the RCT found that treating with CPAP for 4 hours per night or longer was associated with lower incidence of hypertension and CVE (adjusted IDR 0.69 [0.50,0.94]) [21]. Buchener et al. reported a decrease in risk (adjusted HR 0.36 [0.21,0.62]) of overall CVE associated with the use of CPAP [23] and Doherty et al. also found that newly developed hypertension and CVE are significantly less common in treated group [22].

**Fig 2:**
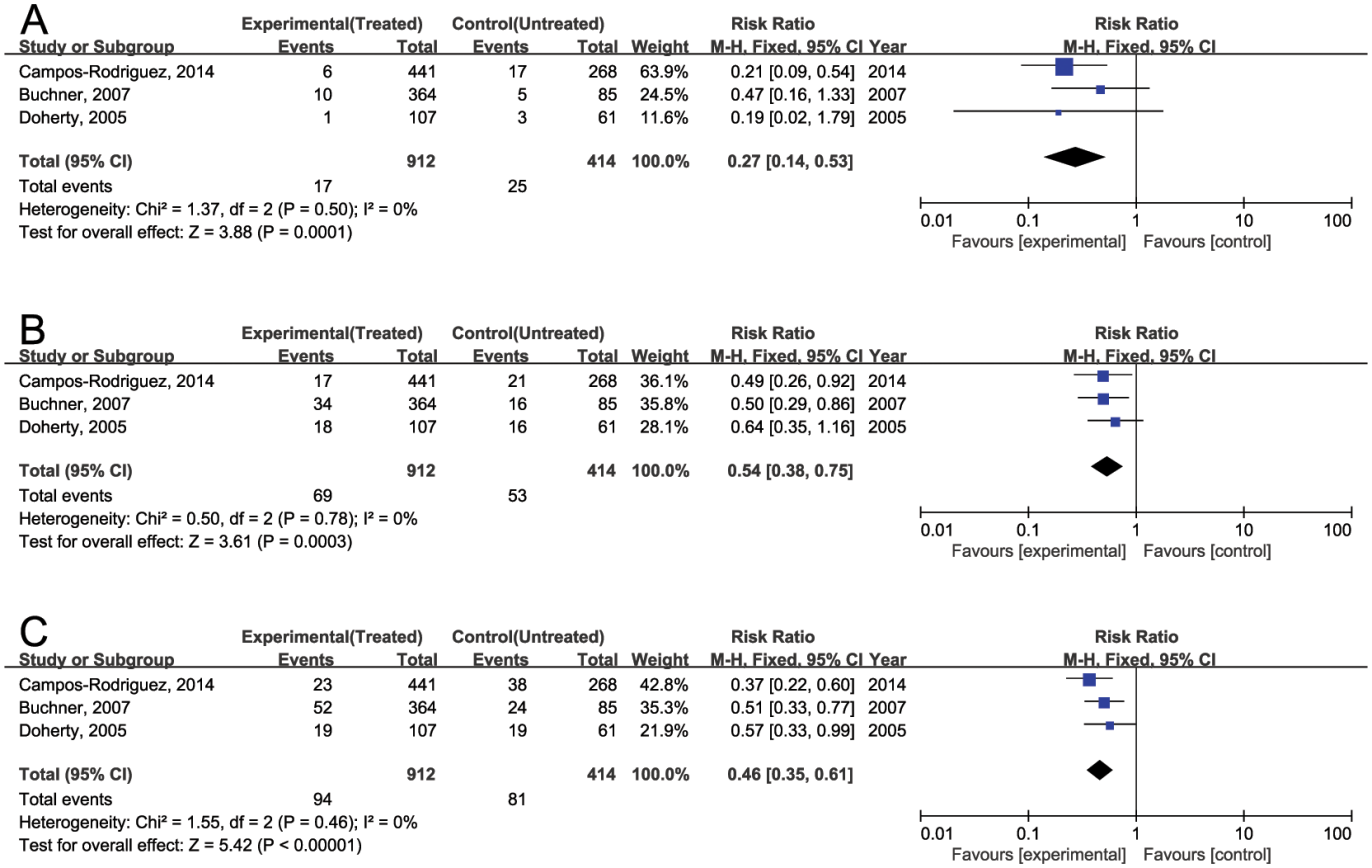
Forest plots of the incidence of CVE. (A) Incidence of stroke. (B) Incidence of cardiac disease. (C) Incidence of overall CVE. Data were calculated by a random-effects model. The boxes represent standardized mean differences (SMDs), and lines depict 95% CIs. The vertical solid line represents no difference between CPAP and control. Values to the right of the solid line favor CPAP benefit. Pooled SMDs and 95% CIs are represented by the diamond shapes.

A meta-analysis from 3 cohort studies showed a decrease in risk with CPAP with an RR 0.27 [0.14, 0.53] for stroke, 0.54 [0.38, 0.75] for cardiac events, and 0.46 [0.35, 0.61] for overall CVE (Fig 2)[22, 23, 26]. There was no significant heterogeneity between the studies (Q statistic, P = 0.46, I^2^ = 0%).

#### Effects on mortality associated with stroke

We identified 3 studies that provided the number of deaths caused by stroke depending on whether CPAP was used or not [22, 24, 25]. Two studies, one which focused on cardiovascular mortality in women and the other in the elderly [24, 25] were from a same cohort with the women cohort study which reported incidence of CVE including both fatal and nonfatal cases [26]. The RCT and Buchner’s cohort study provided the number of death from overall CVE without classification into stroke or cardiac events.

Only the elderly cohort study analyzed the effects of CPAP on mortality from stroke apart from cardiac diseases. Untreated severe OSA was associated with an increased risk of death from stroke (HR 4.63 [1.03, 20.8]) and heart failure (HR 3.93 [1.13, 13.65]), but not from myocardial infarction (HR 1.09 [0.37, 3.36]). The untreated moderate OSA group presented a nonsignificant increase (HR 4.25 [0.88, 20.49]; p = 0.07) in mortality from stroke, but not from either heart failure (HR 1.3 [0.26, 6.48]; p = not significant) or myocardial infarction (HR 0.6 [0.17, 2.2]; p = not significant). The risk was normalized in those treated with CPAP for either stroke or heart failure (HR 1.15 [0.24, 5.5], 1.35 [0.39, 4.63], respectively), compared with the controls without OSA [25].

**Fig 3:**
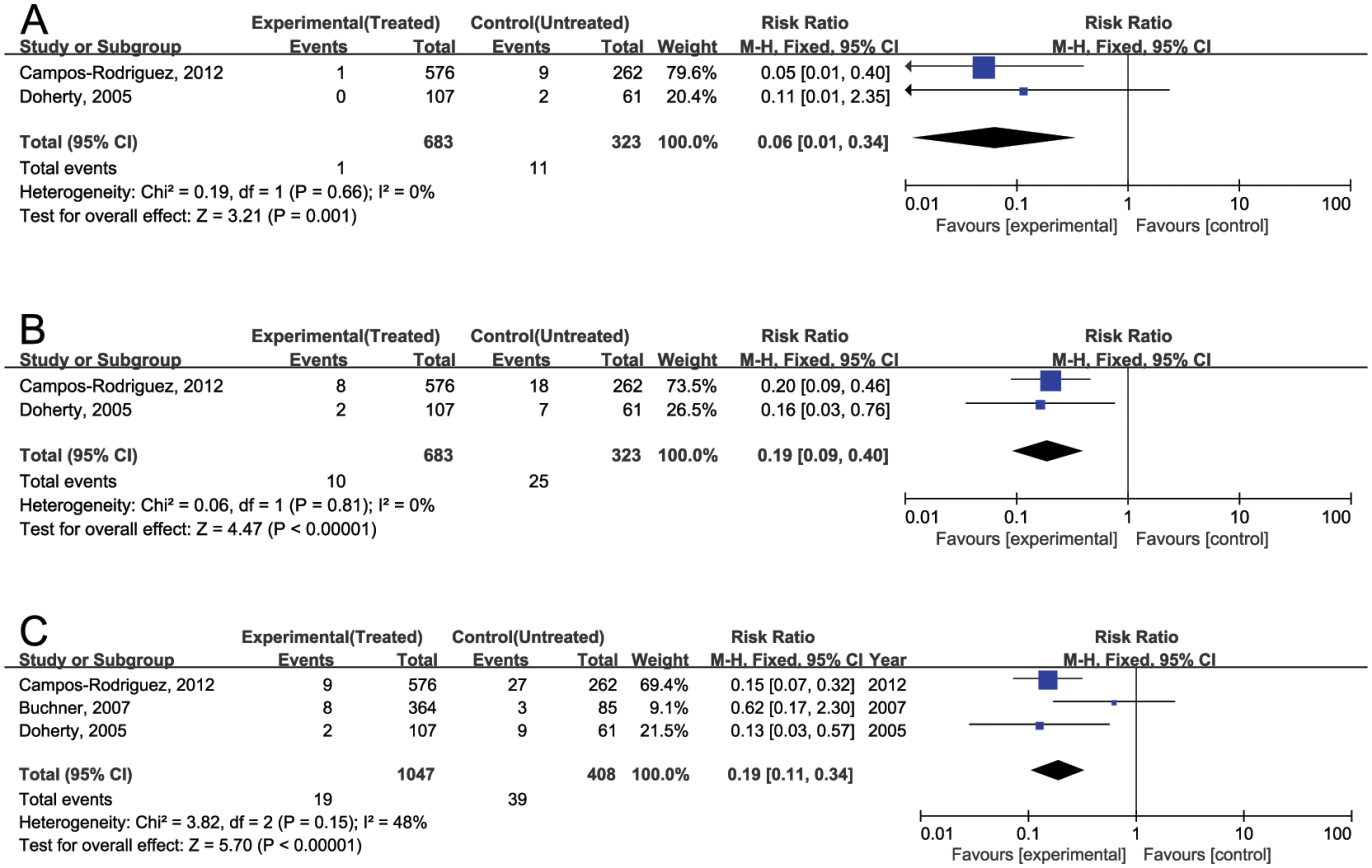
Forest plots of the mortality rates from CVE. (A) Mortality from stroke. (B) Mortality from cardiac disease. (C) Mortality from overall CVE.

A meta-analysis from cohort studies demonstrated a decrease in mortality with CPAP with an RR 0.06 [0.01, 0.34] from stroke, 0.19 [0.09, 0.40] from cardiac events and 0.19 [0.11, 0.34] from overall CVE based on 3 cohort studies [22–24] excepting overlapping studies [25, 26] (Fig 3). The degree of heterogeneity between studies was moderate for overall CVE (Q statistic, P = 0.15, I^2^ = 48%).

#### Factors that may affect the effects of CPAP

None of the studies analyzed how clinical factors might influence the effects of CPAP in stroke apart from the overall CVE.

The risk reduction with CPAP for overall CVE was demonstrated in both women [24, 26] and men, and in both middle-aged patients and the elderly, even those 75 years of age or older [25].

About severity of OSA, three studies included subgroup analyses depending on OSA severity [21, 23, 24]. The RCT couldn’t find a significant effect of CPAP on overall CVE, which was not different across subgroups of AHI or the percentage of time with SaO2 less than 90% [21]. Women cohort study found the reduction of mortality from overall CVE in both groups with AHI of 10–29 and AHI≥30, although the effect was not statistically significant in group with AHI of 10–29 after adjustment [24]. In one of the cohort studies which consisted of predominantly men, a subgroup analysis showed that CPAP reduced the overall CVE also in the group with mild to moderate OSA with an AHI 5 to <30 (adjusted HR 0.37 [0.17–0.78]) as well as those with severe OSA [23].

## Discussion

There are few studies which have investigated the effects of CPAP on stroke in patients with OSA. The cohort studies generally supported the hypothesis that CPAP may reduce the risk of stroke [22–26], whereas the RCT and studies using administrative data did not [21, 27, 28].

The RCT had shorter follow-up period than cohort studies and limited power to assess the effect of CPAP on stroke separately [21]. The studies using administrative data have critical issues of validity and bias. Most of all, administrative database didn’t have the information of sleep study results [27, 28]. CPAP treated group might have more severe OSA than untreated group, which was supported by the finding from the validation sample in Danish study, so the beneficial effect of CPAP could be more pronounced than indicated by those studies [27].

However, biases do exist in cohort studies as well. The untreated group in general consisted of individuals who declined or could not tolerate CPAP [22–26], causing potential bias of better health behavior and better socioeconomic status in the treated group. This might have led to an overestimation of the benefits of CPAP.

On the other hand, baseline differences in the degree of OSA and poor adherence to CPAP might have led to an underestimation the effects of CPAP. The degree of OSA was more severe in most cohort studies [23, 24, 26] and even in the RCT [21]. All of the studies except the Spanish cohort studies [24–26] included individuals with poor adherence to CPAP in the treated group, which may have not truly reflected the efficacy of CPAP. The RCT showed risk reduction of hypertension and CVE in subgroup analysis comparing adherent patients with untreated patients. However, healthy user effect cannot be ruled out in this analysis as well as cohort studies, because the subgroups could not have been randomized based on adherence [21].

Based on a meta-analysis from cohort studies, we found that the treatment with CPAP was associated with lower risk of stroke among patients with OSA with an overall risk reduction of 73%, which was greater than that seen in cardiac disease (46%). This may be due to the difference in mechanisms between stroke and cardiac disease associated with OSA. For example, snoring-associated vibration which may increase carotid artery atherosclerosis [29] and paradoxical embolism in patients with patent foramen ovale during apnea events may be risk factors of stroke but not of cardiac disease [30].

It remains unclear as to whether CPAP has a positive effect on stroke particularly in a certain subgroup. Besides one Spanish cohort study which showed decrease in risk on overall CVE in women[24, 26] and the elderly[25], all the other studies consisted of mostly middle-aged men hence subgroup analysis could not be performed. CPAP seems effective to decrease the risk of overall CVE not only in patients with severe OSA but also those with mild-to-moderate OSA based on a cohort study which consisted of predominantly men [23], but this was not proved in the women cohort study [24]. The association between OSA and stroke or cardiac disease was reported to be stronger in men [7]. The incidence of stroke increased in men with an AHI higher than 19 and women with an AHI higher than 25 [31], and the incidence of coronary disease and heart failure increased in men with OSA but not in women [32]. There was paucity of data for patients younger than 40. In the previous review, the relative mortality rate was highest in their twenties and then decreased with age [33]. This may be due to age-related differences in pathophysiology of OSA or healthy survivor effects [32]. Such phenomena raise the need for analyzing the effects of CPAP for each subgroup. In addition, studies on people outside Europe and US are lacking.

We performed meta-analysis on a limited number of studies that were heterogeneous in terms of inclusion criteria. The number of stroke events was also small and was not shown in detail in each study to allow for any subgroup analysis depending on severity, age, and sex. A larger-scale RCT of longer follow-up should provide more accurate evidence. However, based on the strong evidence for the effect of CPAP on daytime function [34], and some favorable evidence supporting the effects of CPAP on hypertension [3], randomly allocating patients to sham devices for long time could be unethical. For this reason, various strategies are needed to estimate the impact of CPAP on stroke through RCT. The RCT included in this review had studied patients with OSA but without excessive sleepiness. Studying high risk patients could shorten the length of follow-up to show the efficacy as in ongoing RCTs, but obtaining further evidence for primary prevention for stroke from those studies would be challenging. Therefore, in spite of the limitation, this study will provide useful information for treating general people with OSA.

## Conclusion

In spite of the lack of RCTs and somewhat conflicting results among the studies, the data from well-designed cohort studies suggest that CPAP treatment may reduce the risk of stroke in patients with OSA. Such results were more pronounced in stroke than in cardiac disease. Future studies should analyze the effects of CPAP on stroke apart from overall CVE, depending on certain subgroups as according to age, gender, and the severity of OSA.

## Supporting Information

S1 PRISMA Checklist (DOC)

S1 Table The search strategy to search MEDLINE. (DOCX)

## References

[1] Somers VK, White DP, Amin R, Abraham WT, Costa F, Culebras A, et al Sleep apnea and cardiovascular disease: An american heart association/american college of cardiology foundation scientific statement from the american heart association council for high blood pressure research professional education committee, council on clinical cardiology, stroke council, and council on cardiovascular nursing in collaboration with the national heart, lung, and blood institute national center on sleep disorders research (national institutes of health). Journal of the American College of Cardiology. 2008;52(8):686–717. 10.1016/j.jacc.2008.05.002 18702977

[2] Peter B, Ronald G. Positive Airway Pressure Treatment for Obstructive Sleep Apnea-Hypopnea Syndrome In: Meir H K, Thomas R, William C. D, editors. Principles and Practice of Sleep Medicine. 5th ed: Saunders; 2011 p. 1233–49.

[3] Bazzano LA, Khan Z, Reynolds K, He J. Effect of nocturnal nasal continuous positive airway pressure on blood pressure in obstructive sleep apnea. Hypertension. 2007;50(2):417–23. 17548722 10.1161/HYPERTENSIONAHA.106.085175

[4] Benjamin JA, Lewis K. Sleep-disordered breathing and cardiovascular disease. Postgraduate medical journal. 2008;84(987):15–22. 10.1136/pgmj.2007.062836 18230747

[5] Kasai T, Floras JS, Bradley TD. Sleep Apnea and Cardiovascular Disease A Bidirectional Relationship. Circulation. 2012;126(12):1495–510. 10.1161/CIRCULATIONAHA.111.070813 22988046

[6] Yaggi HK, Concato J, Kernan WN, Lichtman JH, Brass LM, Mohsenin V. Obstructive sleep apnea as a risk factor for stroke and death. New England Journal of Medicine. 2005;353(19):2034–41. 16282178 10.1056/NEJMoa043104

[7] Loke YK, Brown JWL, Kwok CS, Niruban A, Myint PK. Association of Obstructive Sleep Apnea With Risk of Serious Cardiovascular Events A Systematic Review and Meta-Analysis. Circulation: Cardiovascular Quality and Outcomes. 2012;5(5):720–8. 22828826 10.1161/CIRCOUTCOMES.111.964783

[8] Mooe T, Franklin KA, Holmstrom K, Rabben T, Wiklund U. Sleep-disordered breathing and coronary artery disease: long-term prognosis. American Journal of Respiratory and Critical Care Medicine. 2001;164(10):1910–3. 11734445 10.1164/ajrccm.164.10.2101072

[9] Elwood P, Hack M, Pickering J, Hughes J, Gallacher J. Sleep disturbance, stroke, and heart disease events: evidence from the Caerphilly cohort. Journal of epidemiology and community health. 2006;60(1):69–73. 16361457 10.1136/jech.2005.039057 PMC2465538

[10] Kernan WN, Ovbiagele B, Black HR, Bravata DM, Chimowitz MI, Ezekowitz MD, et al Guidelines for the prevention of stroke in patients with stroke and transient ischemic attack a guideline for healthcare professionals from the American Heart Association/American Stroke Association. Stroke. 2014;45(7):2160–236. 10.1161/STR.0000000000000024 24788967

[11] Birkbak J, Clark AJ, Rod NH. The effect of sleep disordered breathing on the outcome of stroke and transient ischemic attack: a systematic review. Journal of clinical sleep medicine: JCSM: official publication of the American Academy of Sleep Medicine. 2014;10(1):103. 24426829 10.5664/jcsm.3376 PMC3869059

[12] Furie KL, Kasner SE, Adams RJ, Albers GW, Bush RL, Fagan SC, et al Guidelines for the prevention of stroke in patients with stroke or transient ischemic attack a guideline for healthcare professionals from the American Heart Association/American Stroke Association. Stroke. 2011;42(1):227–76. 10.1161/STR.0b013e3181f7d043 20966421

[13] Parra O, Sánchez-Armengol A, Bonnin M, Arboix A, Campos-Rodríguez F, Pérez-Ronchel J, et al Early treatment of obstructive apnoea and stroke outcome: a randomised controlled trial. European Respiratory Journal. 2011;37(5):1128–36. 10.1183/09031936.00034410 20847081

[14] Martínez-García M, Campos-Rodríguez F, Soler-Cataluña J, Catalán-Serra P, Román-Sánchez P, Montserrat J. Increased incidence of nonfatal cardiovascular events in stroke patients with sleep apnoea: effect of CPAP treatment. European Respiratory Journal. 2012;39(4):906–12. 10.1183/09031936.00011311 21965227

[15] Bassetti CL, Milanova M, Gugger M. Sleep-disordered breathing and acute ischemic stroke diagnosis, risk factors, treatment, evolution, and long-term clinical outcome. Stroke. 2006;37(4):967–72. 16543515 10.1161/01.STR.0000208215.49243.c3

[16] Goldstein LB, Bushnell CD, Adams RJ, Appel LJ, Braun LT, Chaturvedi S, et al Guidelines for the primary prevention of stroke a guideline for healthcare professionals from the American Heart Association/American Stroke Association. Stroke. 2011;42(2):517–84. 10.1161/STR.0b013e3181fcb238 21127304

[17] Moher D, Liberati A, Tetzlaff J, Altman DG. Preferred reporting items for systematic reviews and meta-analyses: the PRISMA statement. Annals of internal medicine. 2009;151(4):264–9. 19622511 10.7326/0003-4819-151-4-200908180-00135

[18] Higgins JP, Altman DG, Gøtzsche PC, Jüni P, Moher D, Oxman AD, et al The Cochrane Collaboration’s tool for assessing risk of bias in randomised trials. Bmj. 2011;343. 10.1136/bmj.d5928 PMC3196245 22008217

[19] Kim SY, Park JE, Lee YJ, Seo H-J, Sheen S-S, Hahn S, et al Testing a tool for assessing the risk of bias for nonrandomized studies showed moderate reliability and promising validity. Journal of clinical epidemiology. 2013;66(4):408–14. 10.1016/j.jclinepi.2012.09.016 23337781

[20] Higgins JPT GSe. Cochrane Handbook for Systematic Reviews of Interventions Version 5.1.0 [updated March 2011] The Cochrane Collaboration, 2011. Available: www.cochrane-handbook.org.

[21] Barbé F, Durán-Cantolla J, Sánchez-de-la-Torre M, Martínez-Alonso M, Carmona C, Barceló A, et al Effect of continuous positive airway pressure on the incidence of hypertension and cardiovascular events in nonsleepy patients with obstructive sleep apnea: a randomized controlled trial. Jama. 2012;307(20):2161–8. 10.1001/jama.2012.4366 22618923

[22] Doherty LS, Kiely JL, Swan V, McNicholas WT. Long-term effects of nasal continuous positive airway pressure therapy on cardiovascular outcomes in sleep apnea syndrome. CHEST Journal. 2005;127(6):2076–84. 10.1378/chest.127.6.2076 15947323

[23] Buchner NJ, Sanner BM, Borgel J, Rump LC. Continuous positive airway pressure treatment of mild to moderate obstructive sleep apnea reduces cardiovascular risk. American Journal of Respiratory and Critical Care Medicine. 2007;176(12):1274–80. 17673692 10.1164/rccm.200611-1588OC

[24] Campos-Rodriguez F, Martinez-Garcia MA, de la Cruz-Moron I, Almeida-Gonzalez C, Catalan-Serra P, Montserrat JM. Cardiovascular mortality in women with obstructive sleep apnea with or without continuous positive airway pressure treatment: a cohort study. Annals of internal medicine. 2012;156(2):115–22. 10.7326/0003-4819-156-2-201201170-00006 22250142

[25] Martínez-García M-A, Campos-Rodríguez F, Catalán-Serra P, Soler-Cataluña J-J, Almeida-Gonzalez C, De la Cruz Morón I, et al Cardiovascular mortality in obstructive sleep apnea in the elderly: role of long-term continuous positive airway pressure treatment: a prospective observational study. American journal of respiratory and critical care medicine. 2012;186(9):909–16. 10.1164/rccm.201203-0448OC 22983957

[26] Campos-Rodriguez F, Martinez-Garcia MA, Reyes-Nuñez N, Caballero-Martinez I, Catalan-Serra P, Almeida-Gonzalez CV. Role of sleep apnea and continuous positive airway pressure therapy in the incidence of stroke or coronary heart disease in women. American Journal of Respiratory and Critical Care Medicine. 2014;189(12):1544–50. 10.1164/rccm.201311-2012OC 24673616

[27] Lamberts M, Nielsen O, Lip G, Ruwald MH, Christiansen CB, Kristensen SL, et al Cardiovascular risk in patients with sleep apnoea with or without continuous positive airway pressure therapy: follow-up of 4.5 million Danish adults. Journal of internal medicine. 2014;276(6):659–66. 10.1111/joim.12302 25169419

[28] Molnar MZ, Mucsi I, Novak M, Szabo Z, Freire AX, Huch KM, et al Association of incident obstructive sleep apnoea with outcomes in a large cohort of US veterans. Thorax. 2015:thoraxjnl-2015-206970. 10.1136/thoraxjnl-2015-206970 PMC4575815 26038534

[29] Lee SA, Amis TC, Byth K, Larcos G, Kairaitis K, Robinson TD, et al Heavy snoring as a cause of carotid artery atherosclerosis. Sleep. 2008;31(9):1207 18788645 PMC2542975

[30] Yaggi H, Mohsenin V. Sleep-disordered breathing and stroke. Clinics in chest medicine. 2003;24(2):223–37. 12800780 10.1016/s0272-5231(03)00027-3

[31] Redline S, Yenokyan G, Gottlieb DJ, Shahar E, O'Connor GT, Resnick HE, et al Obstructive sleep apnea–hypopnea and incident stroke: the sleep heart health study. American Journal of Respiratory and Critical Care Medicine. 2010;182(2):269–77. 10.1164/rccm.200911-1746OC 20339144 PMC2913239

[32] Gottlieb DJ, Yenokyan G, Newman AB, O'Connor GT, Punjabi NM, Quan SF, et al Prospective study of obstructive sleep apnea and incident coronary heart disease and heart failure the sleep heart health study. Circulation. 2010;122(4):352–60. 10.1161/CIRCULATIONAHA.109.901801 20625114 PMC3117288

[33] Lavie P. Mortality in sleep apnoea syndrome: a review of the evidence. European Respiratory Review. 2007;16(106):203–10.

[34] Montserrat JM, Ferrer M, Hernandez L, Farre RN, Vilagut G, Navajas D, et al Effectiveness of CPAP treatment in daytime function in sleep apnea syndrome: a randomized controlled study with an optimized placebo. American Journal of Respiratory and Critical Care Medicine. 2001;164(4):608–13. 11520724 10.1164/ajrccm.164.4.2006034

