# Supplementary material for: Can Continuous Positive Airway Pressure Reduce the Risk of Stroke in Obstructive Sleep Apnea Patients? A Systematic Review and Meta-Analysis
Source: PLoS One. 2016 Jan 5;11(1):e0146317. doi: 10.1371/journal.pone.0146317 (PMC4701420; doi:10.1371/journal.pone.0146317)
Supplement: S1 Table — (DOCX) [file pone.0146317.s002.docx]

**S1. Table. The search strategy to search MEDLINE.**

| theme | number | searches |
| --- | --- | --- |
| A. stroke | 1 | cerebrovascular disorders/ |
|  | 2 | exp basal ganglia cerebrovascular disease/ |
|  | 3 | exp brain ischemia/ |
|  | 4 | exp carotid artery diseases/ |
|  | 5 | stroke/ |
|  | 6 | exp brain infarction/ |
|  | 7 | exp cerebrovascular trauma/ |
|  | 8 | hypoxia-ischemia, brain/ |
|  | 9 | exp cerebrovascular trauma/ |
|  | 10 | hypoxia-ischemia, brain/ |
|  | 11 | exp intracranial arterial diseases/ |
|  | 12 | exp "Intracranial Embolism and Thrombosis"/ |
|  | 13 | exp intracranial hemorrhages/ |
|  | 14 | vasospasm, intracranial/ |
|  | 15 | vertebral artery dissection/ |
|  | 16 | aneurysm, ruptured/ and exp brain/ |
|  | 17 | brain injuries/ |
|  | 18 | brain injury, chronic/ |
|  | 19 | exp carotid arteries/ |
|  | 20 | endarterectomy, carotid/ |
|  | 21 | *heart septal defects, atrial/ or foramen ovale, patent/ |
|  | 22 | (stroke or poststroke or post-stroke or cerebrovasc$ or brain vasc$ or cerebral vasc$ or cva$ or apoplex$ or isch?emi$ attack$ or tia$1 or neurologic$ deficit$ or SAH or AVM).tw. |
|  | 23 | ((brain$ or cerebr$ or cerebell$ or cortical or vertebrobasilar or hemispher$ or intracran$ or intracerebral or infratentorial or supratentorial or MCA or anterior circulation or posterior circulation or basal ganglia) adj5 (isch?emi$ or infarct$ or thrombo$ or emboli$ or occlus$ or hypox$ or vasospasm or obstruction or vasculopathy)).tw. |
|  | 24 | ((lacunar or cortical) adj5 infarct$).tw. |
|  | 25 | ((brain$ or cerebr$ or cerebell$ or intracerebral or intracran$ or parenchymal or intraventricular or infratentorial or supratentorial or basal gangli$ or subarachnoid or putaminal or putamen or posterior fossa) adj5 (haemorrhage$ or hemorrhage$ or haematoma$ or hematoma$ or bleed$)).tw. |
|  | 26 | ((brain or cerebral or intracranial or communicating or giant or basilar or vertebral artery or berry or saccular or ruptured) adj5 aneurysm$).tw. |
|  | 27 | (vertebral artery dissection or cerebral art$ disease$).tw. |
|  | 28 | ((brain or intracranial or basal ganglia or lenticulostriate) adj5 (vascular adj5 (disease$ or disorder or accident or injur$ or trauma$ or insult or event))).tw. |
|  | 29 | ((isch?emic or apoplectic) adj5 (event or events or insult or attack$)).tw. |
|  | 30 | ((cerebral vein or cerebral venous or sinus or sagittal) adj5 thrombo$).tw. |
|  | 31 | (CVDST or CVT).tw. |
|  | 32 | ((intracranial or cerebral art$ or basilar art$ or vertebral art$ or vertebrobasilar or vertebral basilar) adj5 (stenosis or isch?emia or insufficiency or arteriosclero$ or atherosclero$ or occlus$)).tw. |
|  | 33 | ((venous or arteriovenous or brain vasc$) adj5 malformation$).tw. |
|  | 34 | ((brain or cerebral) adj5 (angioma$ or hemangioma$ or haemangioma$)).tw. |
|  | 35 | carotid$.tw. |
|  | 36 | (patent foramen ovale or PFO).tw. |
|  | 37 | ((atrial or atrium or auricular) adj fibrillation).tw. |
|  | 38 | asymptomatic cervical bruit.tw. |
|  | 39 | exp aphasia/ or anomia/ or hemiplegia/ or hemianopsia/ or exp paresis/ or deglutition disorders/ or dysarthria/ or pseudobulbar palsy/ or muscle spasticity/ |
|  | 40 | (aphasi$ or apraxi$ or dysphasi$ or dysphagi$ or deglutition disorder$ or swallow$ disorder$ or dysarthri$ or hemipleg$ or hemipar$ or paresis or paretic or hemianop$ or hemineglect or spasticity or anomi$ or dysnomi$ or acquired brain injur$ or hemiball$).tw. |
|  | 41 | ((unilateral or visual or hemispatial or attentional or spatial) adj5 neglect).tw. |
|  | 42 | apoplexy.tw. |
|  | 43 | 1 or 2 or 3 or 4 or 5 or 6 or 7 or 8 or 9 or 10 or 11 or 12 or 13 or 14 or 15 or 16 or 17 or 18 or 19 or 20 or 21 or 22 or 23 or 24 or 25 or 26 or 27 or 28 or 29 or 30 or 31 or 32 or 33 or 34 or 35 or 36 or 37 or 38 or 39 or 40 or 41 or 42 |
| B. OSA | 44 | exp sleep apnea syndromes/ |
|  | 45 | exp sleep disorders/ and exp respiration/ |
|  | 46 | (sleep$ adj5 (apnea or apnoea or hypopnea or hypopnoea)).tw. |
|  | 47 | (sleep adj5 (cessation or cease$ or periodic) adj5 (respiration or ventilation or air flow)).tw. |
|  | 48 | (SDB or OSAS or CSAS or OSA or SAHS or SAS).tw. |
|  | 49 | snoring.mp. or exp Snoring/ |
|  | 50 | upper airway resistance syndrome.mp |
|  | 51 | exp sleep/ and exp breathing/ |
|  | 52 | 44 or 45 or 46 or 47 or 48 or 49 or 50 or 51 |
| C.CPAP | 53 | exp positive-pressure respiration/ |
|  | 54 | continuous positive airway$.tw. |
|  | 55 | airway pressure release ventilation.tw. |
|  | 56 | (positive pressure or positive-pressure).mp. |
|  | 57 | (CPAP or nCPAP or APRV or biPAP or apap or auto-cpap).mp |
|  | 58 | 53 or 54 or 55 or 56 or 57 or 58 |
| A+B+C | 59 | 43 and 52 and 58 |
